# Forme chronique d'une maladie des éleveurs d'oiseaux: à propos d'une observation

**DOI:** 10.11604/pamj.2015.20.41.3594

**Published:** 2015-01-15

**Authors:** Faten Frikha, Zouhir Bahloul

**Affiliations:** 1 Service de Médecine Interne, CHU Hédi Chaker, 3029 Sfax, Tunisie

**Keywords:** Pneumopathie interstitielle, éleveurs d′oiseaux, tomodensitométrie thoracique, interstitial lung disease, bird breeders, chest CT-scan

## Image en medicine

La maladie du poumon des éleveurs d'oiseaux est une alvéolite d'hypersensibilité secondaire à l'inhalation d'allergène d'origine aviaire (plumes et déjections d'oiseaux). Elle peut se manifester sous 3 formes (aiguë, subaiguë et chronique). Patiente âgée de 54 ans, se présentait pour dyspnée d'effort et toux sèche évoluant depuis une année. L'examen clinique objectivait un hippocratisme digital bilatéral (A). Il existait des râles crépitants à l'auscultation. Le bilan biologique montrait un syndrome inflammatoire, une hyperleucocytose modérée et une hypergammaglobulinémie polyclonale à 15,6 g/L. Le cliché thoracique révélait un syndrome interstitiel avec un aspect flou des 2 champs pulmonaires (B). Le scanner thoracique objectivait une pneumopathie interstitielle diffuse avec un épaississement des lignes septales et des hyperdensités en verre dépoli diffuse (C). L'exploration fonctionnelle respiratoire mettait en évidence un trouble ventilatoire restrictif avec une baisse de la capacité vitale de 35%. La gazométrie artérielle au repos a retrouvé une hypoxémie à 55 mmHg de PaO2. L'interrogatoire poussé nous fait découvrir la notion d’élevage domestique de pigeons et de perroquets depuis 10 ans. Après avoir éliminé les autres causes de pneumopathies interstitielles (biopsie des glandes salivaires accessoires normale, dosage de l'Enzyme de conversion de l'angiotensine normal, enquête infectieuse avec Recherche de BK négative), le diagnostic de la maladie des éleveurs d'oiseaux étaient retenu. La patiente a été traitée par corticothérapie orale (1 mg/Kg/j) avec l’éviction totale de l'exposition. L’évolution était favorable, avec diminution de l'intensité de la dyspnée, de la toux et une stabilisation des lésions radiologiques.

**Figure 1:**
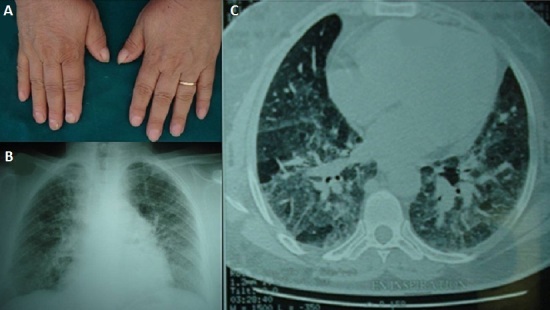
A) aspect d'hippocratisme digital bilatéral; B) radiographie thoracique montrant un syndrome interstitiel bilatéral avec présence de micronodules de faible densité, à contours mal définis; C) tomodensitométrie thoracique: aspect de pneumopathie interstitielle diffuse

